# A Young Female With Thyroid Storm and Pulmonary Embolus: A Case Study

**DOI:** 10.7759/cureus.25690

**Published:** 2022-06-06

**Authors:** William Harper

**Affiliations:** 1 Emergency Department, Trident Medical Center, Charleston, USA

**Keywords:** beta blocker, tachycardia, pulmonary embolism, hyperthyroidism, thyroid storm

## Abstract

Thyroid storm (TS) and pulmonary embolus (PE) are both dangerous conditions. We present a case of a 34-year-old woman suffering from both conditions concomitantly. She was given propranolol, propylthiouracil (PTU), potassium iodide (SSKI), hydrocortisone, and heparin, and improved gradually over the course of a 5-day hospitalization. The patient’s presentation provided difficulties in diagnosis as well as management. Based on our experience with this case, we recommend that the practitioner refrains from prematurely anchoring on one diagnosis without a full workup for the other, as these conditions can be mutually causative. Also, if the patient meets the criteria for TS, it is important to treat them as such, even in the setting of “unimpressive” thyroid study abnormalities. Finally, it is important to administer a beta blocker in the setting of TS, even in the combined setting of PE, as long as the patient has no evidence of heart strain.

## Introduction

Thyroid storm (TS) is a rare but dangerous condition with reported incidences of 0.57-0.76 cases/100,000 persons per year, 4.8-5.6/100,000 hospitalized patients per year in the US, and a mortality rate ranging from 9.5% to 22% [1-4]. Pulmonary embolism (PE) is another dangerous condition, with more recent studies revealing an incidence of 112 cases per 100,000 and a 1-year mortality rate of 13% [5,6]. This case study details the evaluation and care of a patient suffering from PE in the setting of TS. While the concomitant occurrence of these processes has been previously documented, the importance of this particular report lies in the discussion of the difficulties in both diagnosis and management of this patient, the mutually causative relationship between these two conditions, the complicated risk/benefit analysis pertaining to beta blockade in this scenario, and the importance of treating TS aggressively.

## Case presentation

A 34-year-old woman with a history of hyperthyroidism, untreated for the past 5 years, presented to the Emergency Department (ED) via Emergency Medical Services (EMS) with the chief complaint of dyspnea with palpitations. On review of systems, she did endorse loose stools. She denied taking daily medications.

Physical examination revealed: BP 150/78 mmHg; HR 130-170 beats/min; Temperature 36.7 C; respiratory rate 24 breaths/min; and SpO_2_ 100% on room air. The patient was alert and oriented, but hyperactive and with an anxious affect. She repeatedly requested to leave her stretcher and was found pacing back and forth to the bathroom. She was tremulous, with no focal neurologic deficits. With the exception of tachycardia, the cardiovascular exam was within normal limits. Lungs were clear to auscultation. The skin was of normal color. Bowel sounds were hyperactive, but the abdomen was non-tender to palpation.

**Figure 1:**
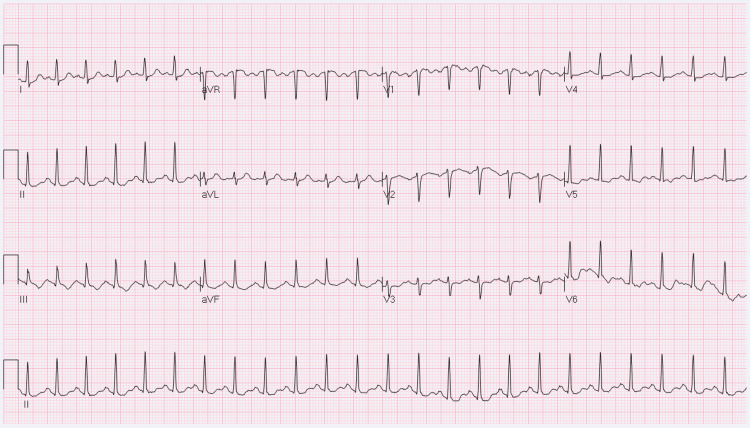
EKG revealing sinus tachycardia at a rate of 140 bpm.

Her initial electrocardiogram (EKG) revealed tachycardia at 170 beats/min that improved to 140 beats/min following lorazepam 1 mg IV for anxiolysis (Figure 1).

Laboratory testing included D-dimer, as PE must be considered in a patient with dyspnea, palpitations, and tachycardia. Basic metabolic panel (BMP), complete blood count (CBC), and troponin were unremarkable, but some lab abnormalities were noted (Table 1).

**Table 1:** Laboratory analyses. TSH: thyroid-stimulating hormone; AST: aspartate aminotransferase; ALT: alanine transaminase

Parameter	Value	Units	Reference Values
D-dimer	463	ng/mL	0-229
TSH	<0.03	µIU/mL	0.34-5.60
Free T4	2.6	ng/dL	0.6-1.1
AST	43	units/L	<35
ALT	76	Units/L	10-63

**Figure 2:**
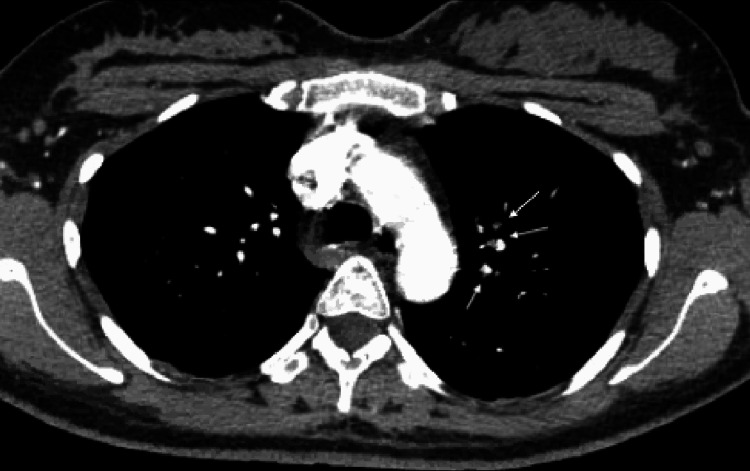
Axial view of CTPA revealing filling defects in the left upper lobe. CTPA: computed tomography pulmonary angiogram

**Figure 3:**
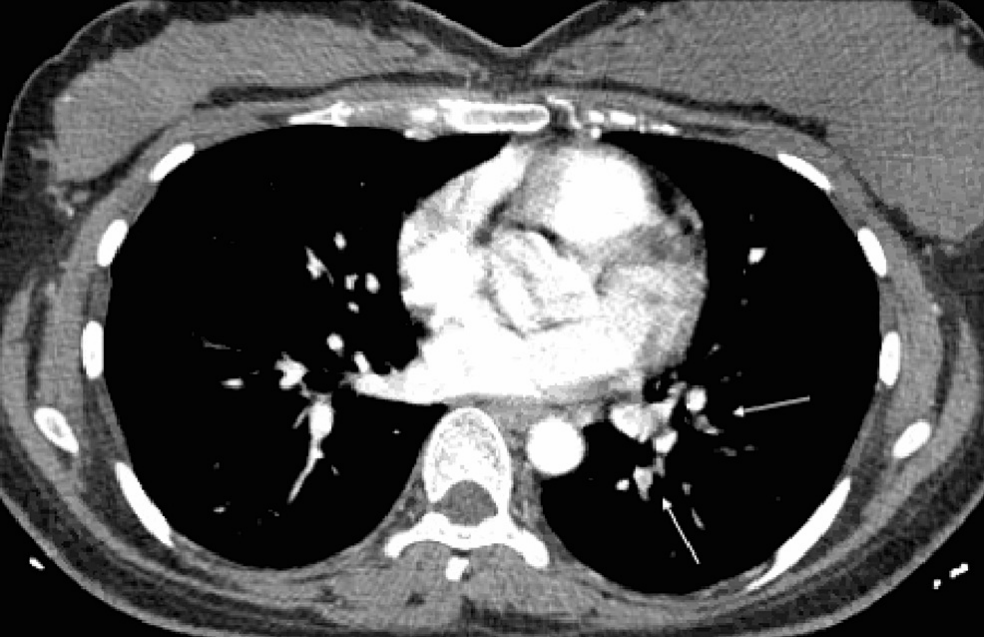
Axial view of CTPA revealing filling defects in the left lower lobe. CTPA: computed tomography pulmonary angiogram

In response to elevated D-dimer, thoracic CT angiogram was performed. This showed multiple branching filling defects (Figures 2-3).

Heparin infusion was initiated. Propylthiouracil (PTU) 200 mg PO (by mouth), hydrocortisone 100 mg IV, lorazepam 1 mg IV, and propranolol 80 mg PO were given. She was admitted to the ICU. Potassium iodide 0.25 g PO was administered 1 hour following PTU. Over 5 days, heart rate and blood pressure improved, and dyspnea, palpitations, hyperactivity, anxiety, and tremors resolved. The patient was discharged on propranolol 60 mg four times daily, PTU 100 mg three times daily, and apixaban 10 mg twice daily.

## Discussion

Concomitant PE and TS present difficulties in both diagnosis and management, with an overlap of signs and symptoms, including elevated temperature, tachycardia, evidence of heart failure, and (in the case of the massive PE) altered mental status. Providers should keep both pathologies in mind when evaluating undifferentiated patients in the ED, even after the initial diagnosis has been determined. In this case, as it became increasingly clear that the patient was suffering from TS, the PE workup was continued, and potentially life-saving treatment was initiated.

Hypercoagulability due to hyperthyroidism has been postulated to be the connection between hyperthyroidism and thromboembolism [7]. Stuijver et al. (2012) concluded that thyroid hormone excess induced a “prothrombotic state,” with increases in factor VIII, factor IX, fibrinogen, von Willebrand factor, and plasminogen activator inhibitor-1 [8]. However, TS is often present due to precipitating processes. A thromboembolic event could act as a precipitant itself, thus creating a mutually causative relationship.

In order to meet the criteria for TS, a patient must have thyroid study abnormalities reflecting hyperthyroidism as well as a Burch and Wartofsky score of 25 or greater. The scale consists of five categories identified as thermoregulatory dysfunction, central nervous system effects, gastrointestinal-hepatic dysfunction, cardiovascular dysfunction, and heart failure. Each category is assigned a score based on severity. If the sum of the points assigned to each category is greater than or equal to 25, the diagnosis of TS is supported. A score of 45 or greater is “highly suggestive” of the TS [9]. This patient’s score was 45.

In this case, the severity of signs and symptoms on presentation was more impressive than the patient’s thyroid study abnormalities. The degree of thyroid hormone abnormality does not significantly differ in patients with TS compared with uncomplicated thyrotoxicosis, making the differentiation based on thyroid hormone levels nearly impossible [10,11]. Therefore, the clinician must aggressively treat all patients meeting criteria for TS.

One challenge in the management of this patient was weighing the benefit of beta blockers in TS with their relative contraindication in PE. Beta blockers (propranolol specifically) alleviate anxiety, heat intolerance, and tachycardia by decreasing adrenergic tone. Propranolol is known to have an inhibitory effect on deiodinase, thereby decreasing the peripheral conversion of T4 to T3 [12]. The beta blockade, however, can be detrimental in patients with compensatory sinus tachycardia in the setting of a submassive or massive PE. CT angiogram of this patient revealed a small embolic load. Additionally, CTA, bedside echocardiogram, and troponin all argued against significant heart strain. A risk-benefit analysis favored the use of propranolol, and the patient responded well.

## Conclusions

If the patient meets criteria for TS, this must be managed aggressively. The practitioner should not be misled by seemingly minor thyroid study abnormalities. Additionally, the present case illustrates the potential peril of anchoring on the diagnosis of one condition, if another condition (PE) has not been appropriately worked up.

Beta blockade is an important aspect of TS management. Without evidence of heart strain, beta blockers should be given. This patient responded well to the indicated beta blockade.
